# Cardiac Remodeling and Functional Changes in Patients With Hypertrophic Cardiomyopathy: A Longitudinal Observational Study

**DOI:** 10.7759/cureus.46610

**Published:** 2023-10-06

**Authors:** Shabarnadh Reddy, Dharma Teja, Rithvika R, Loney Vishal, Harshadeep Gattu, Meghana R Nagilla

**Affiliations:** 1 Department of General Medicine, Narayana Medical College, Nellore, IND; 2 Department of General Medicine, Mamata Medical College, Khammam, IND; 3 Department of General Medicine, Osmania Medical College and Hospital, Hyderabad, IND; 4 Medical School, Rutgers University, Newark, USA

**Keywords:** cardiac function, echocardiography, longitudinal study, cardiac remodelling, hypertrophic cardiomyopathy

## Abstract

Background: Hypertrophic cardiomyopathy (HCM) is a hereditary cardiac disorder characterized by abnormal thickening of the left ventricular myocardium. This can lead to various clinical manifestations, including sudden death.

Aim: To investigate the cardiac remodeling and functional changes in patients with HCM over a specific time period and explore the impact of different treatment regimens on disease progression.

Methods: We conducted a prospective longitudinal observational study involving 100 patients diagnosed with HCM. Baseline clinical data, including demographics, medical history, and echocardiographic measurements, were collected. Follow-up assessments were performed at regular intervals over 24 months to track changes in cardiac structure, function, and clinical status. Statistical analysis, including paired t-tests and subgroup analysis, was conducted to identify significant associations and differences between treatment groups.

Results: A total of 100 patients (mean age = 55 years, 50% male) were enrolled in the study. At baseline, echocardiography revealed increased left ventricular wall thickness (mean = 18.5 mm), left atrial dimensions (mean = 39 mm), and ventricular mass (mean = 230 g). During the follow-up period, there was a progressive increase in left ventricular wall thickness (mean change = 1.0 mm/year, p < 0.001), left atrial dimensions (mean change = 3.0 mm/year, p < 0.001), and ventricular mass (mean change = 8 g/year, p = 0.003). Additionally, alterations in diastolic and systolic function parameters were noted, with a decline in E/A ratio (mean change = -0.1 units/year, p = 0.008) and a reduction in ejection fraction (mean change = -2.0% per year, p = 0.001).

Conclusion: Our longitudinal observational study provides important insights into the cardiac remodeling and functional changes in patients with HCM over time. The progressive increase in cardiac parameters indicates ongoing disease progression. Additionally, beta-blocker therapy was associated with a slower rate of left ventricular wall thickening. These findings contribute to a better understanding of HCM's natural history and may guide targeted therapeutic approaches to improve patient outcomes.

## Introduction

Hypertrophic cardiomyopathy (HCM) is a genetic cardiac disorder characterized by abnormal thickening of the left ventricle myocardium without causative hemodynamic factors, such as hypertension, aortic valve disease, or systemic infiltrative or storage diseases, which affects approximately 0.2-0.5% of the general population [1]. HCM can lead to various clinical manifestations, including dyspnea, chest pain, arrhythmias, heart failure, and sudden death [2,3]. The pathophysiological mechanisms underlying HCM are complex and involve alterations in cardiac structure and function, but the exact disease progression remains poorly understood [4,5].

Several studies have investigated the baseline characteristics and clinical profiles of HCM patients [6-9] but there is limited data on the longitudinal changes in cardiac remodeling and functional parameters over time. Understanding the natural history of HCM and identifying factors associated with disease progression are crucial for optimizing patient management and developing targeted therapeutic interventions.

This study aimed to investigate cardiac remodeling and functional changes in patients with HCM over a specific time period. By tracking changes in echocardiographic parameters and clinical status, we aim to shed light on the disease trajectory and identify potential predictors of adverse outcomes.

## Materials and methods

The study was conducted at Mamata Medical College, Khammam, Telangana, India between January 2020 and July 2023. The study protocol was approved by the Institutional Review Board (IRB) of Narayana Medical College and Hospital (IEC/NMCH/2015/23), and all participants provided written informed consent before enrolment.

Patient recruitment

One hundred patients diagnosed with HCM were recruited from the Cardiology Department of Mamata Medical College, Khammam, Telangana, India. Patients were eligible for inclusion if they met the following criteria: confirmed diagnosis of HCM based on clinical evaluation, electrocardiogram (ECG), and echocardiography, age above 18 years, and ability to provide informed consent for study participation. Exclusion criteria were patients who had an inability to provide informed consent, pregnancy or breastfeeding, and patients with severe comorbidities that could interfere with study participation or interpretation of results. Patients with hypertension, a common condition associated with left ventricular hypertrophy (LVH), were excluded from the study. Additionally, individuals with renal artery stenosis, which can affect blood pressure regulation and contribute to LVH, were not included. Athletes with physiological LVH resulting from intensive physical training were also excluded to isolate cases of pathological LVH. Furthermore, the exclusion criteria encompassed patients with specific cardiac conditions known to cause LVH. These conditions included aortic valve stenosis, coarctation of the aorta, subaortic stenosis, aortic regurgitation, mitral regurgitation, dilated cardiomyopathy, and ventricular septal defect. Patients with any of these conditions were not part of the study. In addition to these cardiac conditions, the exclusion criteria also took into account infiltrative cardiac processes that can lead to LVH, such as amyloidosis, Fabry disease, and Danon disease. Patients with these conditions were ruled out to ensure a clear focus on HCM.

Sample size calculation

The sample size for this study was determined using power analysis. Given the heterogeneity of HCM, we aimed for a study power of 80% and a significance level of 0.05. Based on expected effect sizes, variability, and the need for subgroup analysis, a minimum of 100 patients was targeted.

Distribution across treatment groups

Patients in the study were allocated to treatment groups primarily based on their current medication regimens. This allocation was not randomized but rather determined by the treatments each patient was already receiving at the time of recruitment. Specifically, patients were categorized into the following treatment groups: beta-blocker therapy (n = 50), calcium channel blocker therapy (n = 30), other medications (n = 20), and no medication (n = 15). These categories were established based on the treatments patients were actively using, and no additional criteria were mentioned for allocation.

Data collection

Baseline data were collected at the time of enrolment for each participant. Age, gender, race, and ethnicity were noted along with medical history and demographic variables as follows: along with any recognized cardiac problems, any relatives with HCM, any prior therapies and drugs, and any clinical symptoms, i.e., displaying symptoms such as syncope, palpitations, dyspnea, and chest pain.

Echocardiographic measurements

Echocardiography was performed at baseline to assess cardiac structure and function. Measurements included left ventricular wall thickness, left atrial dimensions, ventricular mass, ejection fraction, and E/A ratio, measured according to the guidelines of the American Society of Echocardiography (2020) [10]. Echocardiographic measurements were obtained using standard techniques by experienced sonographers or cardiologists who were blinded to the patients' clinical status. To ensure the accuracy and reliability of the measurements, a random sample of echocardiograms (10% of the total) was independently reviewed by a second cardiologist. Any discrepancies were resolved through consensus to maintain data integrity. We performed all echocardiographic assessments using the GE Vivid E9 cardiovascular ultrasound system (GE HealthCare, Chicago, IL). This advanced system enabled precise measurements of left ventricular wall thickness, left atrial dimensions, and ventricular mass, ensuring the consistency and reliability of our results throughout the study.

Left ventricular outflow tract obstruction evaluation

The evaluation of left ventricular outflow tract (LVOT) obstruction, a likely cause of symptoms in patients with HCM, was conducted during echocardiographic assessments. Echocardiographic measurements were obtained using standard techniques by experienced sonographers or cardiologists who were blinded to the patients' clinical status.

Pressure gradient measurement

Measurement of the pressure gradient between the LVOT and aorta was performed as part of the echocardiographic evaluation. This critical parameter was assessed to identify and quantify any obstruction or stenosis within the LVOT, which could contribute to symptoms and guide clinical management decisions. The measurements were obtained following established guidelines and protocols.

Treatment regimens

Patients' current treatment regimens, including medications (e.g., beta-blockers and calcium channel blockers) and other interventions, were documented.

Follow-up assessments

Follow-up assessments were conducted at regular intervals, preferably every six months, to track changes in cardiac parameters and clinical status. During each follow-up visit, patients underwent echocardiography and clinical evaluation. Any changes in treatment regimens or disease status were documented.

Statistical analyses were performed using STATA version 16.0 software (StataCorp LLC, College Station, TX). Descriptive statistics were used to summarize demographic characteristics and baseline echocardiographic measurements. Changes in cardiac parameters over time were analyzed using paired t-tests or Wilcoxon signed-rank tests, depending on the distribution of the data. Subgroup analyses were conducted to assess the impact of different treatment regimens on cardiac remodeling and functional changes using the statistical test analysis of variance (ANOVA).

## Results

A total of 100 patients (mean age = 55 years, 50% male) were enrolled in the study. At baseline, echocardiography revealed increased left ventricular wall thickness (mean = 18.5 mm), left atrial dimensions (mean = 39 mm), and ventricular mass (mean = 230 g) (Table 1).

**Table 1:** Baseline characteristics

Parameters	Baseline (mean ± standard deviation)
Age (years)	55 ± 10
Gender (male %)	50%
Left ventricular wall thickness (mm)	18.5 ± 3.0
Left atrial dimensions (mm)	39 ± 4
Ventricular mass (grams)	230 ± 40
E/A ratio	1.5 ± 0.2
Ejection fraction (%)	65 ± 5

During the follow-up period (mean follow-up duration = 24 months), significant changes in cardiac parameters were observed. There was a progressive increase in left ventricular wall thickness (mean change = 1.0 mm/year, p < 0.001), left atrial dimensions (mean change = 3.0 mm/year, p < 0.001), and ventricular mass (mean change = 8 g/year, p = 0.003). Additionally, alterations in diastolic and systolic function parameters were noted, with a decline in the E/A ratio (mean change = -0.1 units/year, p = 0.008) and a reduction in ejection fraction (mean change = -2.0% per year, p = 0.001) (Table 2).

**Table 2:** Changes in cardiac parameters during follow-up

Parameters	Follow-up (mean change ± standard deviation)	p-value
Left ventricular wall thickness (mm)	1.0 ± 0.5	<0.001
Left atrial dimensions (mm)	3.0 ± 1.0	<0.001
Ventricular mass (grams)	8 ± 5	0.003
E/A ratio	-0.1 ± 0.05	0.008
Ejection fraction (%)	-2.0 ± 1.0	0.001

Subgroup analysis based on treatment regimens revealed the following findings.

Beta-blocker therapy

Patients on beta-blocker therapy (n = 50) exhibited a slower rate of left ventricular wall thickening compared to those without beta-blocker therapy (n = 50). The mean left ventricular wall thickness change in patients on beta-blockers was 0.7 mm/year (p = 0.021). The mean left ventricular wall thickness change in patients without beta-blockers was 1.3 mm/year (p < 0.001). Given the possibility of patients being on combination therapy with beta-blockers (BB) and other medications, it is indeed possible that some patients receive a combination of treatments.

Calcium channel blocker therapy

Patients on calcium channel blockers (n = 30) did not show a significant difference in the rate of left ventricular wall thickening compared to those not on calcium channel blockers (n = 70). The mean left ventricular wall thickness change in patients on calcium channel blockers was 1.1 mm/year (p = 0.054). The mean left ventricular wall thickness change in patients without calcium channel blockers was 1.0 mm/year (p < 0.001).

Other medications

A class I antiarrhythmic medication used to manage symptoms like chest pain and palpitations. It can also reduce left ventricular outflow tract obstruction in obstructive HCM.

Amiodarone

An antiarrhythmic drug used to control irregular heart rhythms, particularly atrial fibrillation, which can be associated with HCM.

Anticoagulants/Antiplatelets

Depending on the presence of atrial fibrillation or other risk factors, anticoagulants like warfarin or antiplatelets like aspirin may be prescribed to reduce the risk of blood clots and stroke.

Diuretics

Diuretics like furosemide may be used to manage fluid retention and symptoms of heart failure, particularly in cases of severe HCM.

Statins

These drugs may be considered if there are coexisting high cholesterol levels and atherosclerosis risk factors.

Patients on other medications (n = 20) did not show a significant difference in the rate of left ventricular wall thickening compared to those not on other medications (n = 80). The mean left ventricular wall thickness change in patients on other medications was 1.2 mm/year (p = 0.089). The mean left ventricular wall thickness change in patients without other medications was 1.0 mm/year (p < 0.001).

No medication

A subgroup of patients who did not receive any medication (n = 15) showed a slightly higher rate of left ventricular wall thickening compared to those on medications (n = 85). The mean left ventricular wall thickness change in patients not on medication was 1.3 mm/year (p = 0.072) (Table 3).

**Table 3:** Subgroup analysis based on treatment regimens

Treatment regimens	Left ventricular wall thickness (mm)	p-value
Beta-blocker therapy	0.7 ± 0.3	0.021
No beta-blocker therapy	1.3 ± 0.4	<0.001
Calcium channel blockers	1.1 ± 0.6	0.054
No calcium channel blockers	1.0 ± 0.4	<0.001
Other medications	1.2 ± 0.5	0.089
No other medications	1.0 ± 0.4	<0.001
No medication	1.3 ± 0.7	0.072

## Discussion

The results of our longitudinal observational study on cardiac remodeling and functional changes in patients with HCM provide valuable insights into the natural history of the disease. We observed significant changes in cardiac parameters over the follow-up period, indicating ongoing cardiac remodeling and functional alterations in HCM patients. Our study demonstrated a progressive increase in left ventricular wall thickness, left atrial dimensions, and ventricular mass over time. These findings align with previous research that has consistently reported myocardial hypertrophy as a hallmark of HCM [11]. The thickening of the left ventricular wall can lead to reduced ventricular compliance and impaired diastolic function, contributing to the clinical symptoms observed in HCM patients [12]. We observed a decline in the E/A ratio and a reduction in ejection fraction during the follow-up period. The decreased E/A ratio indicates impaired diastolic function, which is often seen in HCM patients due to increased left ventricular stiffness [13]. The reduction in ejection fraction highlights the progressive impairment of systolic function, indicating a deterioration in the heart's ability to pump blood efficiently. These findings are consistent with previous studies, which have documented diastolic and systolic dysfunction as important features of HCM [11-13].

Our study's findings align with and reinforce the existing literature on cardiac remodeling and functional changes in patients with HCM [14,15]. Consistent with prior research, we observed a progressive increase in left ventricular wall thickness, left atrial dimensions, and ventricular mass over the follow-up period, indicative of ongoing myocardial hypertrophy. These findings are in line with studies conducted by Houston et al. [14], Maron et al. [15], and Masri et al. [16], which also reported similar trends in cardiac remodeling in HCM patients. Moreover, alterations in diastolic and systolic function parameters, exemplified by a decline in the E/A ratio and a reduction in ejection fraction, were noted in our study. These functional changes are consistent with the observations made by Maron et al. [17], who extensively reviewed the management of HCM and highlighted the significance of assessing diastolic and systolic function to understand disease progression.

Our study's comprehensive longitudinal design and inclusion of 100 HCM patients allowed for robust statistical analyses, reinforcing the findings reported by Jacoby DL et al. [18], who emphasized the importance of long-term follow-up and risk stratification in HCM management. Additionally, our subgroup analysis based on different treatment regimens provided further insights into the impact of specific medications on disease progression. Our findings suggested that beta-blocker therapy may be associated with a slower rate of left ventricular wall thickening, consistent with the study by Eriksson et al. [19], which demonstrated improved outcomes in patients with apical HCM treated with beta-blockers.

In conclusion, our study's results align with the existing literature and contribute to the growing body of knowledge on cardiac remodeling and functional changes in HCM patients. By providing longitudinal data in a sizable cohort, our findings strengthen the evidence base for understanding disease progression and may aid in guiding targeted therapeutic approaches to improve patient outcomes, as highlighted by Maron et al. [15]. Overall, our study advances our understanding of HCM's natural history and highlights the significance of continued research in this complex cardiac disorder.

Our subgroup analysis based on treatment regimens revealed that patients receiving beta-blocker therapy exhibited a slower rate of left ventricular wall thickening compared to those without beta-blocker therapy. While beta-blockers have been commonly used in HCM management to control heart rate and improve symptoms, the specific impact on cardiac remodeling has not been extensively studied in a longitudinal context. Our findings suggest that beta-blocker therapy may exert a beneficial effect on reducing myocardial hypertrophy in HCM patients. Our study compared the effects of different medication subgroups on cardiac remodeling [11]. We found that patients on calcium channel blockers or other medications did not show significant differences in the rate of left ventricular wall thickening compared to those not on these medications. However, patients not receiving any medication demonstrated a slightly higher rate of wall thickening compared to those on medications. These observations may have implications for treatment decisions in HCM patients and warrant further investigation to understand the impact of various medications on disease progression.

Our study has some limitations, including the relatively short follow-up duration and the absence of a control group. Additionally, the impact of certain medications might have been confounded by various patient-specific factors. Future research with longer follow-up periods and larger, more diverse patient populations could further elucidate the effects of different treatment regimens on cardiac remodeling and functional changes in HCM.

## Conclusions

In conclusion, our longitudinal observational study provides important insights into the cardiac remodeling and functional changes in patients with HCM over time. The findings are consistent with existing literature, and we also observed the potential beneficial effects of beta-blocker therapy on cardiac remodeling. These results contribute to a better understanding of HCM's natural history and highlight avenues for future research and targeted therapeutic approaches to improve patient outcomes.
